# The miRNAs and their regulatory networks responsible for pollen abortion in Ogura-CMS Chinese cabbage revealed by high-throughput sequencing of miRNAs, degradomes, and transcriptomes

**DOI:** 10.3389/fpls.2015.00894

**Published:** 2015-10-21

**Authors:** Xiaochun Wei, Xiaohui Zhang, Qiuju Yao, Yuxiang Yuan, Xixiang Li, Fang Wei, Yanyan Zhao, Qiang Zhang, Zhiyong Wang, Wusheng Jiang, Xiaowei Zhang

**Affiliations:** ^1^Institute of Horticulture, Henan Academy of Agricultural SciencesZhengzhou, China; ^2^Key Laboratory of Biology and Genetic Improvement of Horticultural Crops, Ministry of Agriculture, Institute of Vegetables and Flowers, Chinese Academy of Agricultural SciencesBeijing, China; ^3^College of Life Science, Zhengzhou UniversityZhengzhou, China

**Keywords:** miRNAs, *Brassica rapa* ssp. *pekinensis*, Ogura-CMS, bud, pollen, deep sequencing

## Abstract

Chinese cabbage (*Brassica rapa* ssp. *pekinensis*) is one of the most important vegetables in Asia and is cultivated across the world. Ogura-type cytoplasmic male sterility (Ogura-CMS) has been widely used in the hybrid breeding industry for Chinese cabbage and many other cruciferous vegetables. Although, the cause of Ogura-CMS has been localized to the orf138 locus in the mitochondrial genome, however, the mechanism by which nuclear genes respond to the mutation of the mitochondrial orf138 locus is unclear. In this study, a series of whole genome small RNA, degradome and transcriptome analyses were performed on both Ogura-CMS and its maintainer Chinese cabbage buds using deep sequencing technology. A total of 289 known miRNAs derived from 69 families (including 23 new families first reported in *B. rapa*) and 426 novel miRNAs were identified. Among these novel miRNAs, both 3-p and 5-p miRNAs were detected on the hairpin arms of 138 precursors. Ten known and 49 novel miRNAs were down-regulated, while one known and 27 novel miRNAs were up-regulated in Ogura-CMS buds compared to the fertile plants. Using degradome analysis, a total of 376 mRNAs were identified as targets of 30 known miRNA families and 100 novel miRNAs. A large fraction of the targets were annotated as reproductive development related. Our transcriptome profiling revealed that the expression of the targets was finely tuned by the miRNAs. Two novel miRNAs were identified that were specifically highly expressed in Ogura-CMS buds and sufficiently suppressed two pollen development essential genes: *sucrose transporter SUC1* and *H*^+^*-ATPase 6*. These findings provide clues for the contribution of a potential miRNA regulatory network to bud development and pollen engenderation. This study contributes new insights to the communication between the mitochondria and chromosome and takes one step toward filling the gap in the regulatory network from the orf138 locus to pollen abortion in Ogura-CMS plants from a miRNA perspective.

## Introduction

Cytoplasmic male sterility (CMS) is a maternally inherited trait that results in the loss of the ability to produce fertile pollen; CMS has been used extensively for hybrid crop breeding (Woodson and Chory, 2008; Luo et al., 2013). Ogura-CMS was originally discovered in the wild radish (*Raphanus sativus*) (Ogura, 1968) and has been widely introduced into Cruciferous vegetables and oil crops and successfully applied in the heterosis breeding industry due to its complete male sterility and stability (Pelletier et al., 1983). Ogura-CMS is controlled by a mitochondrial orf138 locus that consists of two co-transcribed open reading frames: orf138 and atp8 (ATP synthase, subunit 8) (Krishnasamy and Makaroff, 1993; Grelon et al., 1994). However, the mechanism by which the orf138 locus results in male sterility is unclear. Many researchers have focused on the crosstalk between the mitochondria and nucleus, and the CMS is believed to be controlled by mitochondrial-nuclear interactions (Chase, 2007). Several CMS-associated nuclear genes, such as those related to programmed cell death (PCD) and reactive oxygen species (ROS), have been identified and proposed to play roles in the CMS pathway (Balk and Leaver, 2001). Other regulatory signaling pathways, such as the biogenesis of jasmonic acid, are also impaired in the CMS line (Liu et al., 2012).

MicroRNAs (miRNAs) are a class of 21–24 nt small non-coding RNAs that regulate gene expression by post-transcriptional repression (Carrington and Ambros, 2003; Bartel, 2004). In plants, miRNAs are generated from primary miRNA transcripts (pri-miRNAs) that are generally transcribed by RNA polymerase II (Bartel, 2004; Khraiwesh et al., 2010). Primary transcripts containing a distinctive hairpin structure are first trimmed by DICER-LIKE1 (DCL1) to generate miRNA precursors (pre-miRNAs) in the nucleus. Then, these pre-miRNAs are transported to the cytoplasm and further processed by DCL1 to generate ~21 nt mature miRNAs (Kurihara and Watanabe, 2004). Subsequently, the mature miRNAs are loaded onto the RNA-induced silencing complex (RISC) and guide RISC recognition of complementary sites on target mRNAs, thereby inducing transcript cleavage (Bartel, 2004; Baulcombe, 2004) or translational repression (Aukerman and Sakai, 2003; Chen, 2004). Plant miRNAs mainly function by directing the cleavage of their highly complementary target transcripts. Thus, it is easy to predict and verify their targets using bioinformatics and experimental methods.

miRNAs play important roles in the regulation of a wide range of plant developmental processes, including plant architecture (Zhang et al., 2011b), leaf development (Floyd and Bowman, 2004; Juarez et al., 2004), root development (Guo et al., 2005), tuberization (Bhogale et al., 2014), the vegetative-reproductive phase change (Wang et al., 2011a), flowering time (Zhou et al., 2013), floral organ identity (Aukerman and Sakai, 2003; Bartel, 2004; Chen, 2004; Nagpal et al., 2005), self-incompatibility (Tarutani et al., 2010), plant nutrient homeostasis (Yamasaki et al., 2007; He et al., 2014), and response to environmental biotic and abiotic stresses (Juarez et al., 2004; Navarro et al., 2006; Sunkar et al., 2007; Katiyar-Agarwal and Jin, 2010; Zhang et al., 2011a). However, few miRNAs have been confirmed to control pollen development or CMS. Efforts have been made to screen for pollen-specific or enriched miRNAs in rice (Wei et al., 2011), Arabidopsis (Chambers and Shuai, 2009; Grant-Downton et al., 2009a,b), and pakchoi (Jiang et al., 2014).

Chinese cabbage (*Brassica rapa* ssp. *pekinensis*) is an important leafy vegetable that is a cross-pollinated crop with significant heterosis. Ogura-CMS was transferred to the Chinese cabbage in the 1980s and is still widely used in the breeding industry (Yamagishi and Bhat, 2014). The miRNAs from the Ogura-CMS and the maintainer line buds of a closely related subspecies (*B. rapa* ssp. *chinensis*) have been profiled (Jiang et al., 2014). In that study, 54 new conserved miRNAs and 25 pairs of novel miRNAs were identified. Among them, 18 miRNAs were differentially expressed between the male sterile and fertile lines. A genome-wide analysis of miRNAs in *B. rapa* by deep sequencing has been reported (Kim et al., 2012). However, to the best of our knowledge the miRNAs that underlie flower bud development and respond to the Ogura-CMS in Chinese cabbage have not been examined.

In present study, the miRNAs from the buds of a Chinese cabbage Ogura-CMS line (Tyms) and its maintainer line (231-330) were profiled by small RNA deep sequencing. The targets of the miRNAs were identified by degradome sequencing analysis. The expression levels of the corresponding targets were monitored by transcriptome sequencing. These results provide a full set of miRNA-target messages that underlie flower bud development in the Chinese cabbage and provide insights into miRNA-related regulatory functions that may play roles in the mitochondrial–nuclear interactions in Ogura-CMS.

## Materials and methods

### Plant materials and RNA extraction

The Chinese cabbage Ogura-CMS sterile line (Tyms) and its maintainer line (231-330) used in this study were grown in the Henan Academy of Agricultural Sciences, Yuanyang, Henan Province, China. The lines possessed isogenic chromosomes with different cytoplasmic genes. Flower buds < 6 mm length were stripped from 10 individual of each genotype of plants. These buds contain the entire developmental progress from anther generation to pollen abortion. Tyms and 231-330 were sampled separately and then snap-frozen in liquid nitrogen and kept at -80°C for further use. Total RNA was extracted using the TRIzol reagent (Invitrogen, USA). DNase (Promega, USA) was used to remove potential DNA contamination.

### Small RNA library construction and sequencing

The RNA samples from 231-330 (Control) and Tyms (CMS) were quantified and equalized so that equivalent amounts of RNA were analyzed. A total of 30 μg of RNA was resolved on denatured polyacrylamide gels. Gel fragments with the size range of 18–30 nt were excised and recovered. These small RNAs were ligated with 5′ and 3′RNA adapters with the T4 RNA ligase. The adapter-ligated small RNAs were subsequently transcribed into cDNA by Super-Script II Reverse Transcriptase (Invitrogen) and amplified using primers specific for the ends of the adapters. The amplified cDNA products were purified and finally sequenced using Solexa sequencing technology (BGI, Shenzhen, China).

### Identification of known and novel miRNAs

The adapter sequences, impurities, and sequences with less or more than 18–30 nt were filtered out from the raw sequence reads. The remaining sequences that ranged from 18 to 30 nt in length were used for known miRNA prediction. First, we aligned the tags to the *B. rapa* miRNA precursors in miRBase (version 20.0, http://www.mirbase.org/index.shtml) with no mismatches allowed. Then, the obtained tags were aligned to the mature miRNAs of *B. rapa* with at least a 16 nt overlap to allow offsets. The miRNAs that satisfied both of the above criteria were counted to obtain the expression number of identified miRNAs. The rest of the small RNA tags were aligned to the miRNA precursors/mature miRNAs of all plants in miRBase, allowing two mismatches and free gaps. The miRNAs with the highest expression levels for each mature miRNA family were chosen as a temporary miRNA database. Then, the precursors of the temporary miRNAs in the *B. rapa* genome were predicted. Those that failed to fold into hairpin structures were regarded as pseudo-miRNAs and discarded, while those that fulfilled the miRNA criteria were adopted as newly identified known miRNAs.

By comparing our sequences with those in the databases and the Chinese cabbage genome, the sRNAs can be annotated into different categories, including siRNA, piRNA, rRNA, tRNA, snRNA, snoRNA, repeat associated sRNA, degraded tags of exons or introns, and sRNAs that could not be annotated. The tags annotated as intron, exon antisense, and unknown were used to predict novel miRNAs using the software Mireap. The key conditions are as follow: hairpin miRNAs can fold into secondary structures and mature miRNAs are present in one arm of the hairpin precursors; the 5-p and 3-p mature miRNAs present 2-nucleotide 3′ overhangs; hairpin precursors lack large internal loops or bulges; the secondary structures of the hairpins are steady, with the free energy of hybridization lower than or equal to −18 kcal/mol; and the copy number of mature miRNAs with predicted hairpins must be greater than five in the alignment result. The expression of novel miRNAs was produced by summing the count of miRNAs with no more than three mismatches on the 5′ and 3′ ends and no mismatches in the middle from the alignment result.

The differentially expressed miRNAs were calculated with the following procedures. First, the expression of miRNAs in two samples was normalized to obtain the expression of transcript per million (TPM). The normalization formula was as follows: Normalized expression = Actual miRNA count/Total count of clean reads × 1,000,000; Second, the fold-change and *P*-value were calculated from the normalized expression.

### Degradome library construction and target identification

Total RNA extracted from the 231-330 (Control) and Tyms (CMS) lines. Approximately 200 μg of the total RNA was polyadenylated using the Oligotex mRNA mini kit (Qiagen). A 5′ RNA adapter was added to the cleavage products (which possessed a free 5′-monophosphate at their 3′ termini) using the T4 RNA ligase (Takara). Then, the ligated products were purified using the Oligotex mRNA mini kit (Qiagen) for reverse transcription to generate the first strand of cDNA using an oligo dT primer via SuperScript II RT (Invitrogen). After the cDNA library was amplified for 6 cycles (94°C for 30 s, 60°C for 20 s, and 72°C for 3 min) using Phusion Taq (NEB), the PCR products were digested with the restriction enzyme Mme I (NEB). A double-stranded DNA adapter was ligated to the digested products using T4 DNA ligase (NEB). The ligated products were selected based on size in a 10% polyacrylamide gel and purified for the final PCR amplification (94°C for 30 s, 60°C for 20 s, and 72°C for 20 s) for 20 cycles (German et al., 2009). The PCR products were gel purified and used for high-throughput sequencing with the Illumina HiSeq 2000.

Low quality sequences and adapters were removed, and the unique sequence signatures were aligned to the database of Chinese cabbage transcript assemblies in the Brassica Gene Index (http://brassicadb.org/brad/downloadOverview.php) using the SOAP software (Li et al., 2008) (http://soap.genomics.org.cn/). CleaveLand was used to detect potentially cleaved targets based on degradome sequences. The 20 and 21 nt distinct reads were subjected to the CleaveL and pipeline for small RNA target identification as previously described (Addo-Quaye et al., 2009). The tags mapped to cDNA sense strands were used to predict cleavage sites. The miRNA-mRNA pairs were searched and *p*-values were calculated using PAREsnip (http://srna-workbench.cmp.uea.ac.uk/tools/paresnip/). Only *p*-values less than 0.05 were adopted for the *t*-plot figure (Folkes et al., 2012). All alignments with scores not exceeding 4 that possessed the 5′ end of the degradome sequence coincident with the 10th and 11th nucleotides that were complementary to the small RNA were retained. To evaluate the potential functions of miRNA-targeted genes, gene ontology (GO) categories (http://www.geneontology.org/) were used to assign the identified target genes according to the previously described method (Du et al., 2010).

### Transcriptome sequencing and target gene profiling

Total RNA (10 μg) was subjected to poly-A selection, fragmentation, random priming, first and second strand cDNA synthesis with the Illumina Gene Expression Sample Prep kit (CA, USA). The cDNA fragments were subjected to an end repair process and then ligated to adapters. The products were enriched with PCR, and the 200-bp fragments were purified with 6% TBE PAGE gel electrophoresis. After denaturation, the single-chain fragments were fixed onto the Solexa Sequencing Chip (Flowcell) and consequently grown into single-molecule cluster sequencing templates through in situ amplification on the Illumina Cluster Station. Double-end pyrosequencing was performed on the Illumina Genome Analyzer platform with read lengths of 101 bp for each end. The clean reads were aligned to the Chinese cabbage reference transcript assemblies (http://brassicadb.org/brad/downloadOverview.php). Gene expression levels were calculated using the RPKM method (Mortazavi et al., 2008) using the following formula: RPKM = (1,000,000 ^*^ C)/(N ^*^ L ^*^ 1000), where RPKM(A) is the expression of gene A, C is the number of reads that uniquely align to gene A, N is the total number of reads that uniquely align to all genes, and L is the number of bases in gene A. Statistical comparison between Tyms and 231–330 was performed using the IDEG6 software (Romualdi et al., 2003). The General Chi-squared method was used and the FDR (false discovery rate) was applied to determine the Q-value threshold. Unigenes were considered to be differentially expressed when the RPKM between Tyms and 231–330 displayed a more than two-fold change with an FDR less than 10^−2^.

### Quantitative real-time PCR

For analysis of miRNAs, 2.5 μg of total RNA was polyadenylated using a miRNA cDNA synthesis kit (Takara, Inc., Dalian, China). The poly(A)-tail-amended total RNA was reverse-transcribed by PrimeScript RTase using a universal adapter primer containing oligo-dT. The qPCR was performed on a LightCycler 96 System (ROCHE, USA) using SYBR Premix Ex TaqTMII (TaKaRa, Dalian China). The miRNA-specific forward primer for each miRNA was designed based on the entire miRNA sequence (Table S13), and the universal reverse primer was provided by the miRNA cDNA synthesis kit (Takara, Dalian, China). All reactions were performed with three biological and three technical replicates for each sample, and the U6 snRNA (Forward: GGGGACATCCGATAAAATT, Reverse: TGTGCGTGTCATCCTTGC) was used as the internal control. The reaction volume was 20 μL, including 10 μL of SYBR *Premix Ex Taq* II, 0.8 μL of 10 mM Forward primer, 0.8 μL of 10 mM Uni-miR qPCR primer, 2.0 μL of the cDNA sample and 6.4 μL of dH_2_O. The following qPCR program was used: denaturation at 95°C for 30 s, followed by 40 cycles of 95°C for 5 s, 55°C for 30 s, and 72°C for 60 s. Melting curve analysis with 95°C for 10 s, 65°C for 60 s, and 97°C for 1 s was performed to produce a dissociation curve for verification of the amplification specificity. Relative expression levels of miRNAs were quantified using the 2^−Δ*ΔCt*^ method (Livak and Schmittgen, 2001).

The primers of the selected genes subjected to target analysis are listed in Table S14. β*-actin* was used as an internal control. Experiments were performed on a similar system as described above. The reaction volume was 20 μL, including 10 μL of SYBR® *Premix Ex Taq*™ (Tli RNaseH Plus), 0.8 μL of 10 mM Forward primer, 0.8 μL of 10 mM Reverse primer, 2.0 μL of the cDNA sample and 6.4 μL of dH_2_O. Three independent biological and three technical replicates were performed. The fold change was estimated using the 2^−Δ*ΔCT*^ method (Livak and Schmittgen, 2001).

### Paraffin sectioning and microscopic observation

Sterile and fertile flower buds were fixed and embedded in Paraffin (Beeswax, China). Thin (0.8 μm) sections were prepared with an Ultracut Eultra microtome (Leica, Germany), stained with hematoxylin, and photographed under a LEICA DMI3000B microscope (Leica, Germany).

## Results and discussion

### sRNA sequencing and miRNA identification

**Figure 1:**
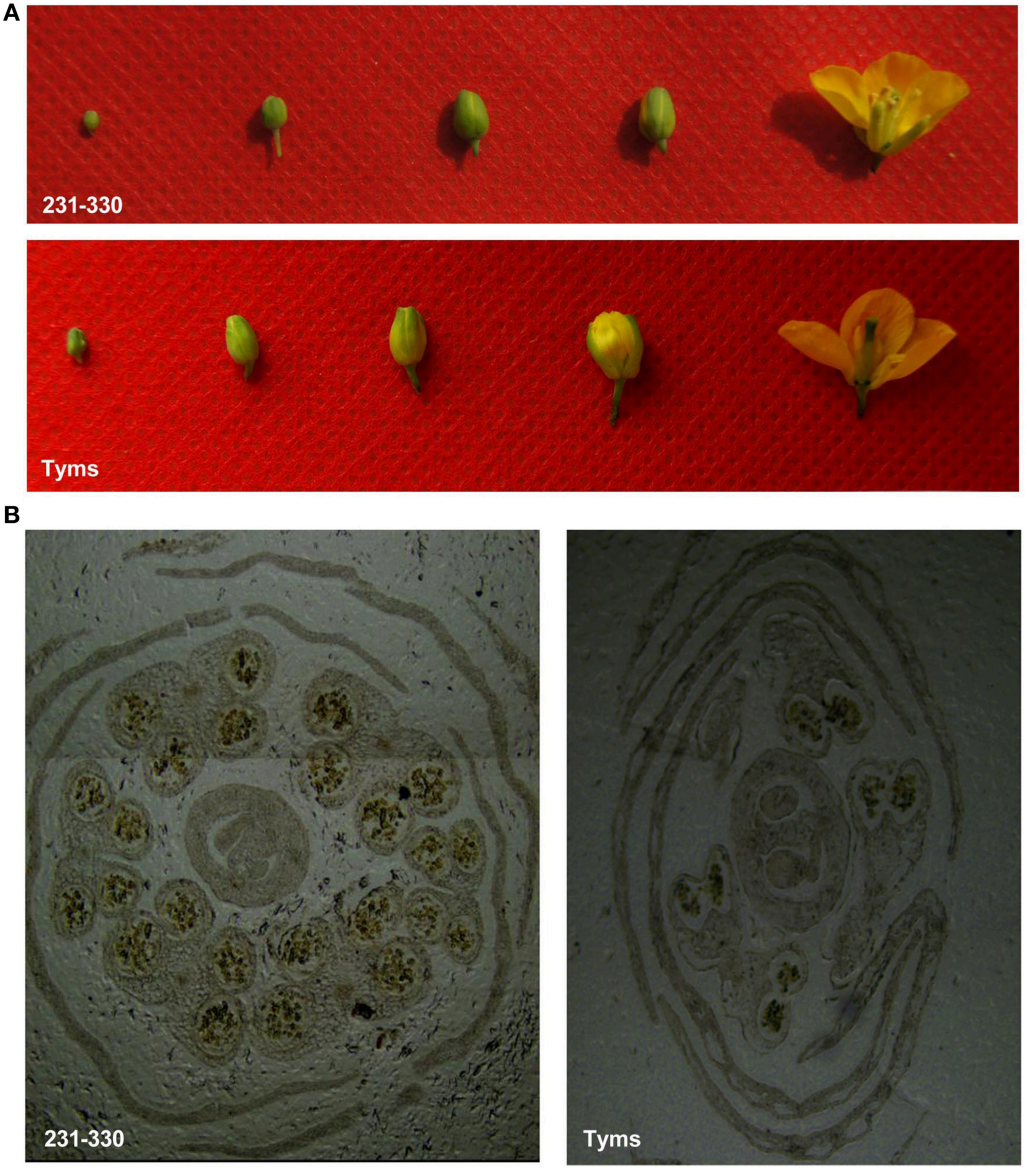
**The buds and flowers of the Ogura-CMS (Tyms) and maintainer (231-330) lines. (A)** the buds of four developmental stages and the flower that were sampled for this study. **(B)** microscopy of the buds showing the well-developed pollen sacs in 231–330 and incomplete development in Tyms.

**Figure 2:**
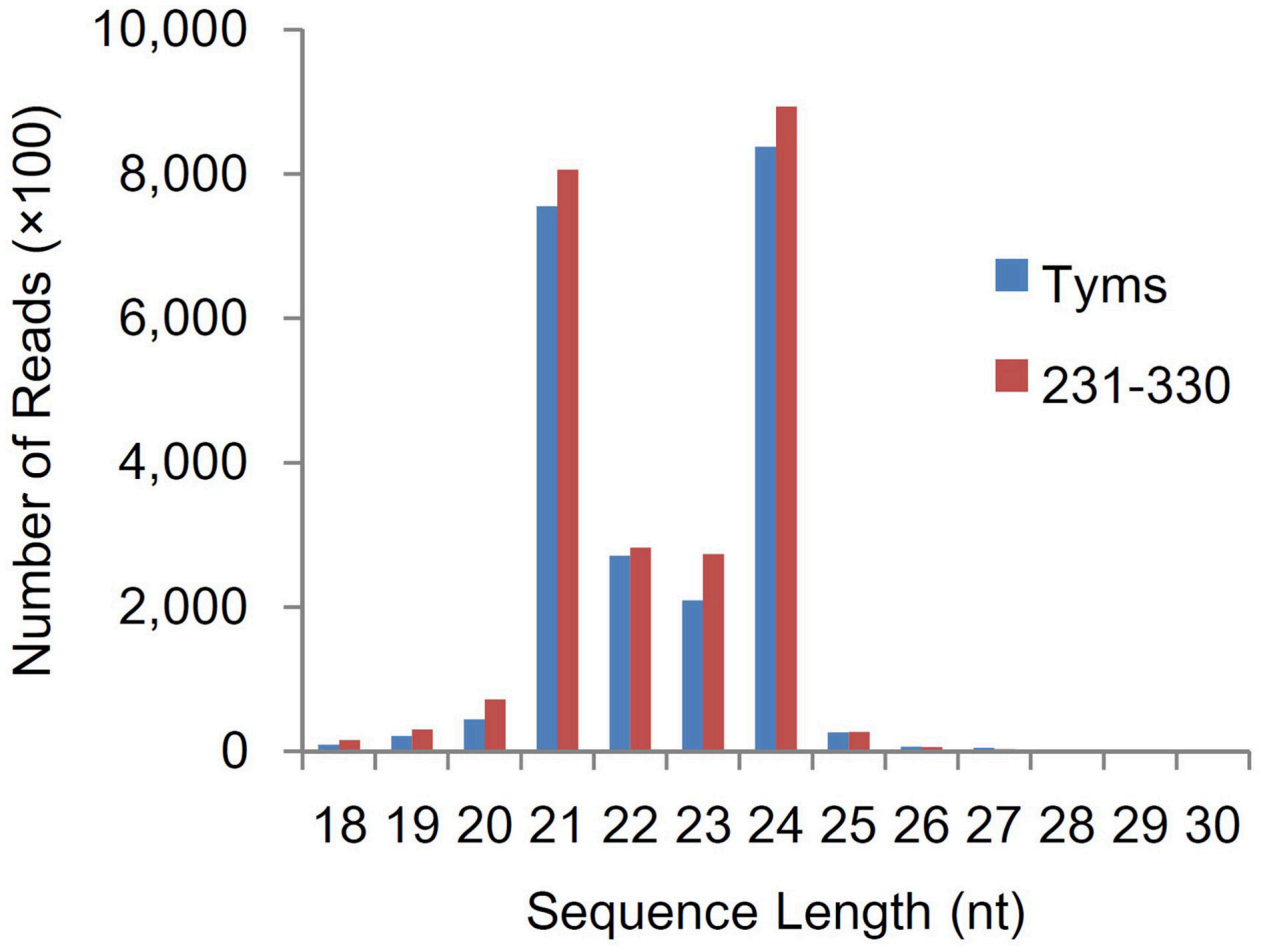
**Size distribution of small RNA sequences**.

To analyze the roles of miRNAs in Ogura-CMS in Chinese cabbage, small RNAs were pyrosequenced from the buds of the Ogura-CMS Tyms line and its maintainer line 231–330 (the morphology and microscopy of the samples are shown in Figure 1). A total of 22.2 and 24.4 M reads were produced from the two lines, respectively. After filtering out the low quality reads, 3′adapter null, insert null, 5′adapter contaminants, reads smaller than 18 nt and poly A, a total of 21.9 and 24.1 M high quality clean reads were obtained, respectively (Table S1). The majority of the tags ranged in size between 21–24 nt, with the 24 and 21 nt lengths dominant (Figure 2). Out of the 46.0 M total tags, 33.8 M (73.5%) were shared by the two samples, while 5.7 M (12.5%) and 6.4 M (14.0%) were Tyms- and 231–330-specific, respectively. Then, the tags were divided into 12.2 M non-redundant unique reads composed of 4.8 M (39.6%) Tyms-specific, 5.6 M (45.7%) 231–330-specific and 1.8 M (14.8%) reads shared by both lines (Table S2). The reads were aligned to *B. rapa* miRNAs (*B. rapa* 1.1) in miRBase (http://www.mirbase.org/); among the 157 mature miRNAs encoded by 96 precursors distributed into 63 families included in the database, 69 mature miRNAs from 59 precursors of 41 families were expressed in our samples (Table S3). The remainder of the reads were aligned to all known plant miRNAs in miRBase 21.0 and then aligned to the *B. rapa* genome for precursor identification. From this analysis, another 220 mature miRNAs derived from 163 precursors were identified. Among them, 57 precursors contained both 5p and 3p miRNAs, while only one mature miRNA was detected for the remaining 106 precursors. The 139 (39 pairs and 61 singles) mature miRNAs encoded by 100 precursors were new members of 36 existing families in the *B. rapa* miRBase. Thus, only 17 families (bra-MIR9552—bra-MIR9557 and bra-MIR9559—bra-MIR9569) in the miRBase *B. rapa* collection were not detected in our present study. By expending the members to the existing *B. rapa* miRNA families, bra-miR156 was now the largest family (harboring 21 members), followed by bra-miR171 (11 members), bra-miR167 (8 members), bra-miR172 (8 members), bra-miR164 (7 members), bra-miR168 (7 members), bra-miR2111 (7 members), bra-miR157 (6 members), bra-miR160 (6 members), bra-miR390 (6 members), and bra-miR395 (6 members). The other families contained less than five members (Table S4). In addition to these existing families, we also identified 81 miRNAs (18 pairs and 45 singles from 63 precursors) belonging to 23 families that had not been previously reported in *B.rapa*. These included 13 members of bra-miR1439, seven members of bra-miR169, five members each of bra-miR166, bra-miR393, bra-miR394, and bra-miR399, three bra-miR165, two bra-miR170, two bra-miR5376, and one member each of bra-miR397, bra-miR827, bra-miR828, bra-miR838, bra-miR845, bra-miR858, bra-miR5298, bra-miR5575, bra-miR5641, bra-miR6029, bra-miR6030, bra-miR6033, bra-miR6034, and bra-miR6284 (Table S5). The remaining reads were aligned to Genbank, Rfam and the cabbage genome and annotated as rRNA, scRNA, snRNA, snoRNA, tRNA, repeat, exon sense, exon antisense, intron sense, intron antisense, and unannotated sequences (Table S6). The unannotated reads and those derived from the intron region and exon antisense region were used for novel miRNA analysis. A total of 426 novel miRNAs were identified based on the criteria defined in the Methods section. The sequences and precursor information were listed in Table S7. Among them, 83 novel miRNAs were generated from more than one precursor in the Chinese cabbage. A total of 138 precursors (84 types of mature miRNA) harbored both the 3-p and 5-p miRNAs on their arms (Supplementary file 2).

Of these miRNAs, members of 32 known families were also identified in buds of a closely related vegetable plant *B. rapa* ssp. *chinensis* (Jiang et al., 2014). These miRNAs may be common bud development contributors in both plants. Our present study identified more miRNAs of either known or novel type than that of the previous report. One important reason for this is that nearly fourfold of reads were generated in present study compared to the previous one. This facilitated the identification of low abundant miRNAs. Another reason can be attributed to the genetic differences between the two subspecies. One of our sequencing projects showed only half of reads from *B. rapa* ssp. *chinensis* could map to the *B. rapa* ssp. *pekinensis* reference genome (unpublished data). The publicly released *B. rapa* ssp. *pekinensis* genome (Wang et al., 2011b) is not an ideal reference for *B. rapa* ssp. *chinensis*. Jiang et al. (2014) used *B. rapa* ssp. *pekinensis* reference genome for their miRNA annotation and novel-miRNA prediction, there for many miRNAs were failed to be identified due to the sequence diversity, especially for the novel miRNAs which rely on the hairpins prediction.

### Comparative expression patterns of miRNAs in the buds of Ogura-CMS and its maintainer

The expression levels of known and novel miRNAs were profiled based on tag counts. For multiple precursors sharing the same mature miRNA, only one member of the mature miRNA was used for expression profiling. The miRNA with the highest expression levels was bra-miR157, which accumulated 579,691 and 253,845 copies in the 231-330 and Tyms buds, respectively, followed by bra-miR168 and bra-miR156 (Table S3). bra-miR156 and bra-miR157 belong to the same family and regulate the *SQUAMOSA promoter binding protein-like* (*SPL*) genes that are important regulators for plant vegetative and reproductive development as well as gynoecium differential patterning and male fertility (Wang et al., 2009; Wu et al., 2009; Xing et al., 2010, 2013; Zhang et al., 2011b; Yu et al., 2012). bra-miR168 targets *ARGONAUTE 1* (*AGO1*), which is an important part of the RISC. The balance between miR168 and AGO1 plays important roles in plant development, including flowering and fruiting (Vaucheret et al., 2004; Xian et al., 2014). The top 20 most highly expressed known miRNAs are listed in Table 1. The expression levels of novel miRNAs were much lower than the known miRNAs, with the majority (~80%) barely accumulating several to less than 100 copies. The top 15 most highly expressed novel miRNAs are listed in Table 2.

**Table 1:** **The most highly expressed known miRNAs**.

**miRNA**	**Count**		**Sequence**
	**231-330**	**Tyms**	
bra-miR157	579,691	253,845	TTGACAGAAGATAGAGAGCAC
bra-miR168	506,969	373,990	TCGCTTGGTGCAGGTCGGGAC
bra-miR156	254,093	231,666	TTGACAGAAGAAAGAGAGCAC
bra-miR166	151,210	170,161	TCGGACCAGGCTTCATTCCCC
bra-miR158-5p	66,094	51,736	CTTTGTCTATCGTTTGGAAAAG
bra-miR158-3p	44,804	34,621	TTTCCAAATGTAGACAAAGCA
bra-miR5718	39,028	17,531	TCAGAACCAAACACAGAACAAG
bra-miR408-5p	34,067	32,148	ACAGGGAACAAGCAGAGCATG
bra-miR172b-3p	23,598	16,041	AGAATCTTGATGATGCTGCAT
bra-miR390-5p	21,103	15,941	AAGCTCAGGAGGGATAGCGCC
bra-miR164	15,166	12,237	TGGAGAAGCAGGGCACGTGCA
bra-miR165	14,685	15,120	TCGGACCAGGCTTCATCCCCC
bra-miR6034	12,828	12,514	TCTGATGTATATAGCTTTGGG
bra-miR168-3p	9015	5512	CCCGCCTTGCATCAACTGAAT
bra-miR1885	7209	8016	TACATCTTCTCCGCGGAAGCTC
bra-miR6029	6847	4093	TGGGGTTGTGATTTCAGGCTT
bra-miR160-3p	5180	4304	GCGTATGAGGAGCCATGCATA
bra-miR5654	4807	3418	ATAAATCCCAAGCATCATCCA
bra-MIR5721	4345	3606	AAAATGGAGTGGGAAATGGAG
bra-miR393-3p	4325	4780	ATCATGCGATCTCTTCGGATT

**Table 2:** **The most highly expressed novel miRNAs**.

**miRNA**	**Sequence**	**Count**		
		**231-330**	**Tyms**	**Total**
novel-miR-154	GGAATGTTGTCTGGCTCGAGG	17,293	17,185	34,478
novel-miR-113	GTCTGGGTGGTGTAGTCGGTT	3337	1862	5199
novel-miR-269	GGACTGTTGTCTGGCTCGAGG	2362	1902	4264
novel-miR-3	GCGTACAGAGTAGTCAAGCATA	1790	1949	3739
novel-miR-310	TTAGCGGAATATAAGAATCGGTT	2398	1127	3525
novel-miR-228	CTTGCATATCTTAGGAGCTTT	1690	1582	3272
novel-miR-74	TTGCTATAGATGGTTTCTGCT	1781	1340	3121
novel-miR-287	AGATCATCCTGCGGCTTCATT	1152	1336	2488
novel-miR-275	TGAAGTGGAGTAGAGTATAATG	1424	730	2154
novel-miR-121	GGAATGTTGTTTGGCTCGAAG	984	887	1871
novel-miR-199	TGGATGATGCTTGGCTCGAGA	446	1160	1606
novel-miR-320	TGGTAGAACGACCGCATGATC	789	756	1545
novel-miR-50	GCAGCACCATCAAGATTCACA	913	507	1420
novel-miR-261	TCTGCTATCAACTTGTAGAGTC	795	612	1407
novel-miR-55	GCAGCATCATCAAGATTCACA	536	607	1143

The expression level between the Ogura-CMS and maintainer buds was compared using normalized tag counts. Ten known miRNAs were down-regulated two-fold (*p* < 10^−3^) in Tyms compared to 231–330 (Table 3). Among them, bra-miR157, bra-miR158-3p, and bra-miR5718 were expressed at relatively high levels and could play important roles in pollen development. However, bra-miR6030 was the only known miRNA that was up-regulated in the Tyms buds. In addition to these examples, a total of 49 and 27 novel miRNAs were down- and up-regulated in Tyms, accounting for 11.5 and 6.3% of the total 426 novel miRNAs, respectively (Table S8).

**Table 3:** **Differentially expressed known miRNAs**.

**Pairwise**	**miR-name**	**231-330-std**	**Tyms-std**	**Fold-change(log2 Tyms/231-330)**	***p*-value**	**Change**
231−330−Tyms	bra−miR157	24058.52	11589.58	−1.05	0	Down
231−330−Tyms	bra−miR158−3p	494.17	79.76	−2.63	0	Down
231−330−Tyms	bra−miR169	58.73	27.53	−1.09	3.62E−59	Down
231−330−Tyms	bra−miR394	20.00	9.63	−1.05	4.01E−20	Down
231−330−Tyms	bra−miR827	19.76	8.67	−1.19	9.70E−24	Down
231−330−Tyms	bra−miR845	22.33	11.09	−1.01	7.63E−21	Down
231−330−Tyms	bra−miR858	11.91	5.07	−1.23	9.91E−16	Down
231−330−Tyms	bra−miR5714	1.83	0.82	−1.15	3.21E−3	Down
231−330−Tyms	bra−miR5716	3.44	1.37	−1.33	5.17E−06	Down
231−330−Tyms	bra−miR5718	1619.75	800.40	−1.02	0	Down
231−330−Tyms	bra−miR6030	7.64	20.55	1.43	1.11E−32	Up

**Figure 3:**
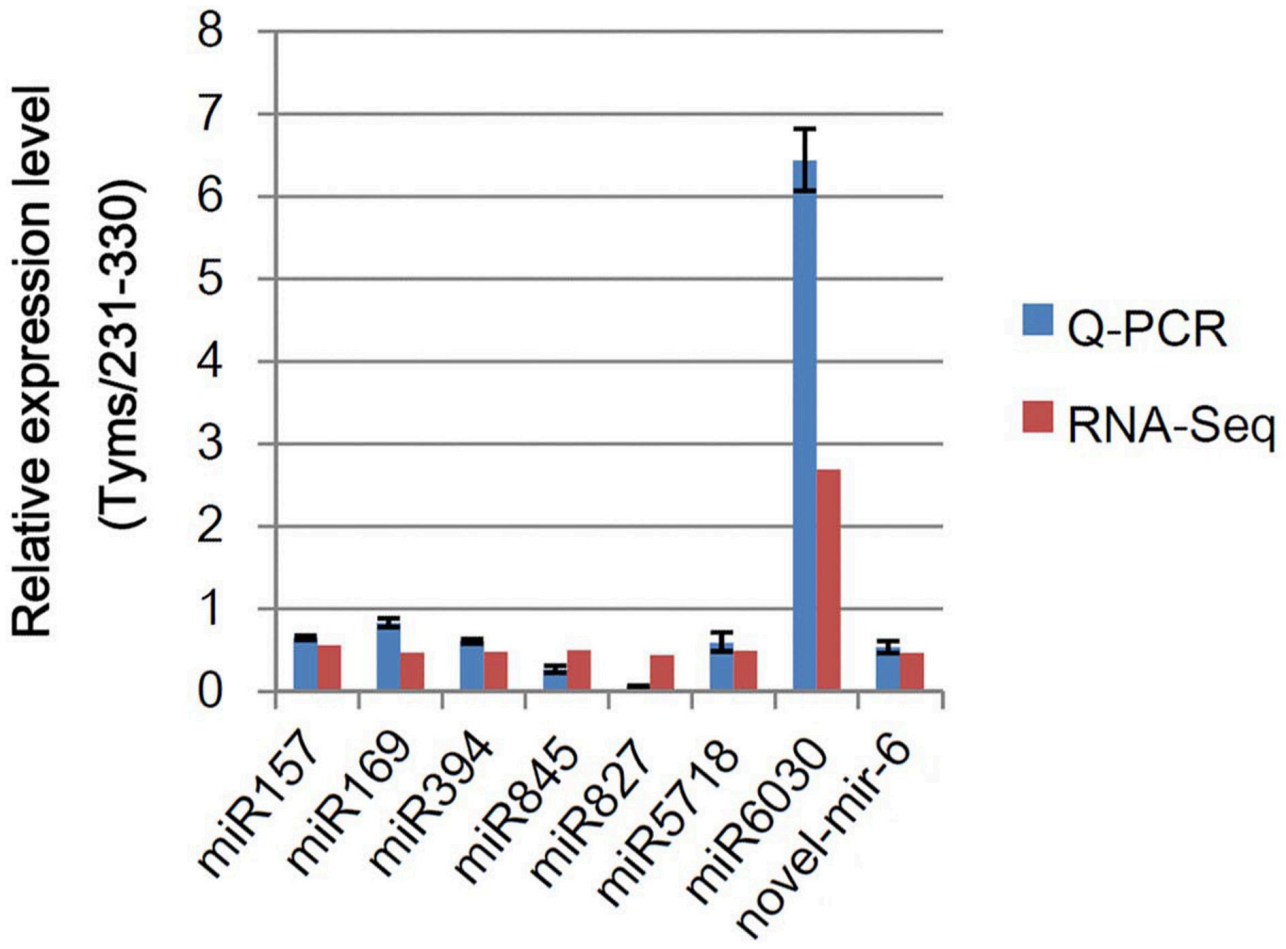
**Q-PCR analysis of the relative expression levels of miRNAs**. The bars indicate the SE; four biological and three technique replicates were performed.

To test the accuracy of the RNA-Seq-based expressional profiling, a set of Q-PCR analyses were performed. As shown in Figure 3, the relative expression levels were similar between the Q-PCR and RNA-Seq technologies for seven known and one novel miRNAs. These results indicate that the RNA-Seq expression profiles are reliable.

### Target prediction and validation by degradome analysis

**Figure 4:**
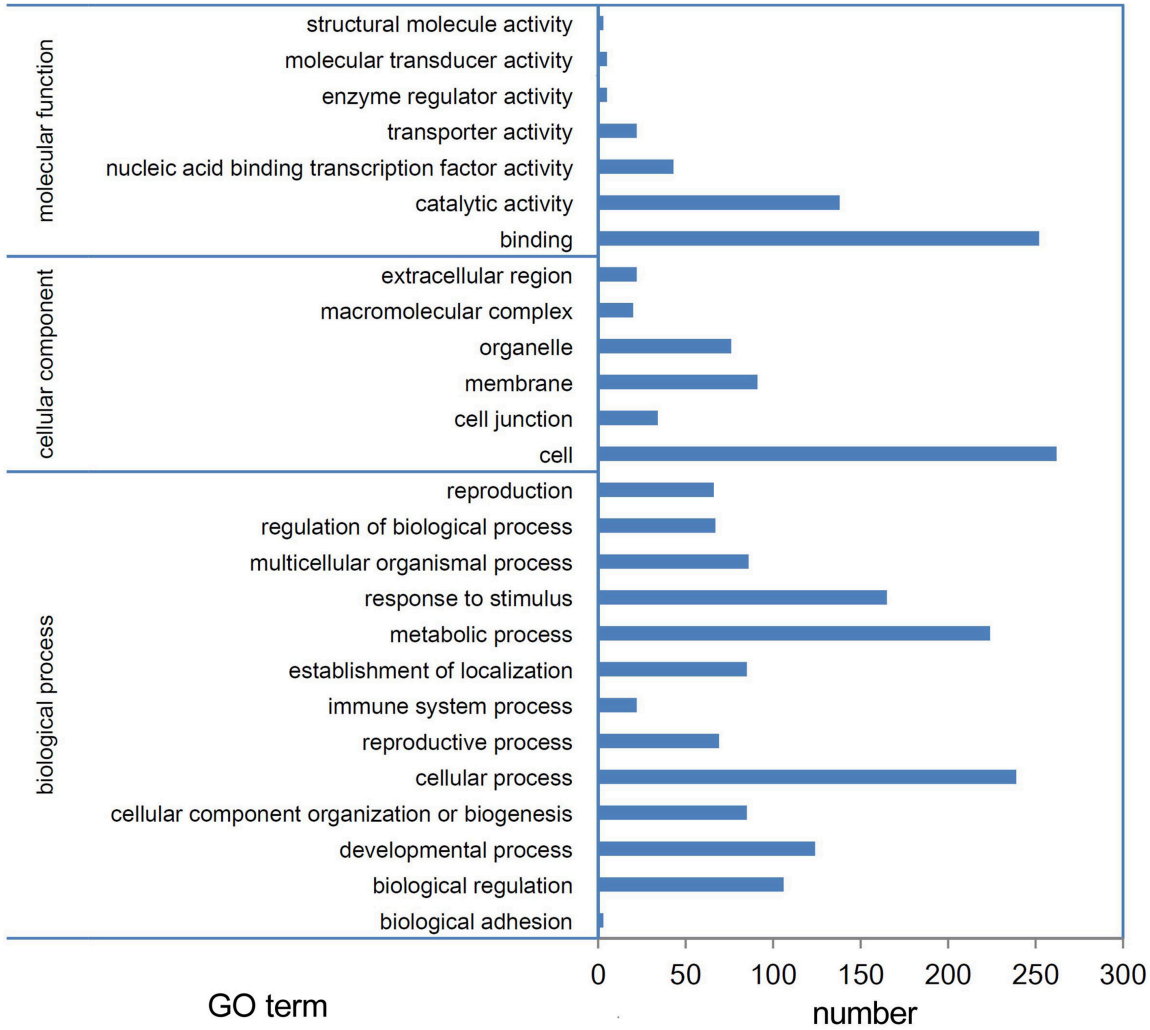
**GO annotation of targets**. Targets of miRNAs expressed in Tyms and 231-330 Chinese cabbage buds were detected by degradome sequencing analysis.

miRNAs function by regulating their target genes and especially by degrading their target mRNAs in plants. Thus, we performed a degradome sequencing analysis to validate the miRNA targets. A total of 36,541,512 and 36,595,548 clean tags were generated from the buds of Tyms and 231-330, respectively (Table S9). Among them, 23,253,666 (63.64%) and 24,768,149 (67.68%) tags were mapped to the reference *B. rapa* genome. A total of 15,154,675 (41.47%) and 17,009,287 (46.48%) tags were mapped to the cDNA sense chains and were used for target prediction (Table S10). A total of 376 mRNAs were identified as miRNA targets, including 136 targeted by 30 known miRNA families and 248 targeted by 100 novel miRNAs. Among which 26 mRNAs were targeted by more than one miRNA (Table S11). A larger proportion known (50.8%) miRNAs compared to novel (23.5%) miRNAs have targets been detected, indicating that the conserved miRNAs have comparatively more valid cutting functions than the newly formed miRNAs. One possible reason for not detecting the targets could be that the target mRNAs were expressed at low levels; thus, the cut ends were not detected by our pipeline. Another possibility is that some miRNAs function by translation inhibition rather than mRNA digestion. Based on GO annotation, the targets were enriched in the “binding,” “catalytic activity,” and “nucleic acid binding transcription factor activity” terms in the “molecular function” cluster. “Cell,” “membrane,” and “organelle” were the three most abundant “cellular component” targets. In the “biological process” cluster, the top three terms were “cellular process,” “metabolic process,” and “response to stimulus” (Figure 4).

Most of the known miRNAs targeted transcription factor-encoding genes, such as the targeting of *SPL, MYB, auxin response factor (ARF), NAC, scarecrow, APETALA 2 (AP2), GROWTH-REGULATING FACTOR (GR)*, and *C3HC4-type RING finger* family transcription factors by miR156/157, miR159, miR160, miR164, miR171, miR172, miR396, and miR5716, respectively. This finding indicates that the transcription factors perform primary important roles in bud development. Another large group of miRNAs, including miR158, miR161, miR400, and miR5654, target the pentatricopeptide repeat (PPR)-containing protein-encoding genes. PPR-containing proteins are a large family in plants that are mostly located in the mitochondria and chloroplast and play important roles in RNA processing in the two organelles (Fujii and Small, 2011). Some PPR-containing proteins have been identified as fertility restoration (Rf) genes for CMS (Desloire et al., 2003; Wang et al., 2008; Yasumoto et al., 2009). miR1885, miR5719, and miR6030 target disease resistance (CC-NBS-LRR class) genes. miR167 targets *IAA-amino acid hydrolase 3* (*IAR3*), which encodes an auxin conjugate hydrolase that contributes to the jasmonate pathway (Kinoshita et al., 2012; Widemann et al., 2013). MiR393 targets *AUXIN signaling F-BOX* (*AFB*) genes, which are auxin receptors that have been widely identified as stress response regulators and control auxin-related development in plants (Si-Ammour et al., 2011; Terrile et al., 2012). These genes and the above mentioned miR160-ARFs indicate that the miRNA-hormone signaling cascades play important roles in flower bud development. Interestingly, three *Vacuolar ATP synthase subunit A* (*VHA-A*) tags were identified as targets of miR5712. *VHA-A* have been demonstrated to be essential for male gametophyte development due to its important role in Golgi organization (Dettmer et al., 2005). miR5724 targets *embryo defective 1473* (*emb1473*), which encodes a structural constituent of the ribosome. miR158, which is down-regulated in Tyms buds, targets a glutathione S-transferase gene that has been annotated as a restorer-of-fertility (Krishnasamy and Makaroff, 1994; Woo et al., 2008). These miRNAs could play important roles in pollen development.

For the novel miRNAs, 153 out of the 264 targets (58%) were annotated as “catalytic activity,” which accounted for the largest fraction of the targets. Only 16 transcription factors were identified as targets of novel miRNAs: four *NAC* (including three *cup-shaped cotyledon*) targeted by novel mir410, 446, and 465, four zinc finger family proteins, two homeobox proteins, one *MYB*, one *WRKY*, one *SPL*, one *HSFA1E*, and one *PIL1* (*Phytochrome Interacting Factor 3-like 1*). Several of the 19 targets possess “transporter activity,” including four genes encoding H^+^-transporting ATPases, two genes encoding Ca^2+^:H^+^ antiporters and two genes encoding sucrose transporter SUC1. These genes could play important roles in gametophyte development by transporting protons, ions, mineral elements and energy. A total of 28 targets were annotated as reproductive process-related, including the aforementioned *cup-shaped cotyledon 2* transcription factors and *sucrose transporter SUC1, auxin-responsive protein*, and *ubiquitin-activating enzyme E1* (Table S12). Among them, an oxidoreductase, a HSFA1E, a C2H2 zinc finger protein 1 and a beta-glucosidase were targets of novel-miR-6, −93, and −321, which were down-regulated in Tyms. An F-box/LRR-repeat protein, a heat shock 70 kDa protein 1/8, an auxin-responsive protein IAA and two sucrose transporter SUC1 were targeted by novel-miR-175, −383, −385, and −448, which were up-regulated in Tyms buds.

### Target expression profiles based on transcriptome sequencing

To profile the expression of the targets, a transcriptome sequencing project was performed separately on buds of 231–330 and Tyms. A total of 25,015,186 and 24,295,576 clean reads were generated from the 231–330 and Tyms buds, respectively. Approximately 76.4 and 72.6% of the reads were mapped to the reference genome and 59.3 and 57.9% of the reads were mapped to the reference gene sets of *B. rapa*, respectively. The expression of the target genes was called from the transcriptome data for further analysis. A total of 367 and 352 out of the 376 target genes were expressed in the 231–330 and Tyms buds, respectively. Among them, 44 were up-regulated and 32 were down-regulated (two-fold change and FDR < 10^−3^) in Tyms compared with 231–330. Most of the differentially expressed targets were pollen development-related genes according to the annotation (Tables 4, 5).

**Table 4:** **The up-regulated targets in Tyms compared to 231-330 buds**.

**geneID**	**RPKM (231–330)**	**RPKM (Tyms)**	**log2 (T/231)**	**FDR**	**Annotation**
Bra011781	0.153	2.406	3.980	1.13E−10	Growth-regulating factor 2J
Bra004474	0.881	8.341	3.243	1.42E−22	Galactinol synthase
Bra006956	0.420	1.767	2.073	4.46E−04	Growth-regulating factor 4
Bra017379	3.814	14.809	1.957	6.43E−16	Lysosomal thiol reductase family protein
Bra012155	3.935	14.720	1.904	2.77E−41	U-box domain-containing protein 2
Bra004363	1.826	6.322	1.791	3.02E−08	Squamosa promoter-binding protein-like 6
Bra004892	62.505	215.114	1.783	0	Transducin family protein
Bra000864	0.256	0.860	1.751	8.10E−04	Ent-copalyl diphosphate synthase
Bra027897	20.504	67.391	1.717	8.67E−123	CYP96A15
Bra028175	42.722	135.558	1.666	3.16E−147	Ribulose bisphosphate carboxylase small chain
Bra017851	0.872	2.751	1.658	3.44E−05	Growth-regulating factor 2P
Bra005019	30.610	96.012	1.649	8.96E−157	RCA (RUBISCO activase)
Bra000096	2.553	7.910	1.631	4.77E−14	Calmodulin-binding protein-like
Bra022685	1.998	5.976	1.580	5.35E−08	Cup-shaped cotyledon2
Bra029216	2.231	6.648	1.575	1.67E−07	LRR-repeat protein
Bra016937	14.438	41.059	1.508	6.99E−34	S-adenosyl-L-methionine-dependent methyltransferase
Bra017143	2.326	6.533	1.490	2.49E−15	Ceramidase family protein
Bra012263	44.469	120.598	1.439	3.82E−68	Zinc-binding family protein
Bra001369	3.402	9.054	1.412	4.47E−09	3-phosphoinositide-dependent protein kinase-1
Bra031329	37.673	98.231	1.383	8.45E−89	Cell wall-plasma membrane linker protein
Bra028181	2288.119	5965.931	1.383	0	Ribulose bisphosphate carboxylase small chain
Bra023844	5.298	13.778	1.379	5.22E−24	CRK10 (cysteine-rich rlk10)
Bra023235	89.500	228.685	1.353	2.52E−232	ATP binding / phosphoribulokinase
Bra034079	2.458	6.219	1.339	2.56E−16	Disease resistance protein
Bra030820	4.925	12.084	1.295	3.46E−10	NAC1
Bra024616	9.169	22.415	1.290	2.68E−21	Oxidoreductase
Bra030727	23.394	56.454	1.271	1.36E−51	Calreticulin-3
Bra013767	2.533	5.912	1.223	9.3E−06	Growth-regulating factor 8
Bra005131	47.935	111.401	1.217	1.35E−112	cax51
Bra030188	6.137	14.006	1.190	4.11E−06	Nudix hydrolase 25
Bra025427	1.529	3.475	1.185	2.49E−04	Brassinosteroid-6-oxidase 1
Bra036417	4.213	9.514	1.175	2.08E−17	Disease resistance protein
Bra013013	15.281	33.823	1.146	1.46E−26	Calcium sensing receptor
Bra012691	2.217	4.896	1.143	5.87E−04	2OG-Fe(II) oxygenase family protein
Bra020262	3.260	7.194	1.142	5.32E−07	TOE2 transcription factor
Bra023066	1.993	4.257	1.095	7.54-04	Growth-regulating factor 4
Bra014184	7.485	15.606	1.060	4.65E−15	Cytochrome c biogenesis protein CCS1
Bra003311	4.714	9.724	1.045	2.85E−09	Scarecrow transcription factor
Bra040873	42.657	87.512	1.037	1.49E−64	Aminoacylase
Bra012008	3.631	7.428	1.033	1.17E−06	Cysteine/histidine-rich C1 domain-containing
Bra036135	8.977	18.315	1.029	7.59E−05	Lysosome-related
Bra011537	56.705	114.452	1.013	9.85E−94	O-Glycosyl hydrolases family 17 protein
Bra013556	3.991	8.044	1.011	7.72E−10	Homeobox-leucine zipper protein HDG2-like
Bra003518	4.133	8.286	1.004	9.55E−09	Transport inhibitor response 1

**Table 5:** **The down-regulated targets in Tyms compared to 231-330 buds**.

**geneID**	**RPKM (231-330)**	**RPKM (Tyms)**	**log2 (T/231)**	**FDR**	**Annotation**
Bra016846	19.369	0.001	−14.241	1.71E−72	SYP125
Bra031759	18.677	0.001	−14.189	6.8E−70	SYP125
Bra019872	12.375	0.162	−6.258	9.21E−43	SYP125
Bra015756	9.216	0.001	−13.170	3.18E−44	Serine/threonine-protein kinase
Bra008330	5.324	0.001	−12.378	2.81E−17	Pleckstrin homology (PH) domain-containing
Bra026884	2.748	0.001	−11.424	7.39E−16	Pentatricopeptide (PPR) repeat-containing
Bra035163	547.451	1.757	−8.284	0	H^+^-ATPase 9
Bra013168	677.150	6.843	−6.629	0	H^+^-ATPase 6
Bra003491	331.459	1.268	−8.030	0	VGDH2 (Vanguard 1 homolog 2)
Bra004481	1608.187	8.885	−7.500	0	Pectinesterase 5
Bra030518	19.054	0.164	−6.864	3.32E−133	Clathrin assembly protein
Bra030371	607.258	10.827	−5.810	0	Late embryogenesis abundant protein−like
Bra005178	226.835	4.629	−5.615	0	Actin 3
Bra028534	28.543	0.607	−5.555	6.96E−138	Purple acid phosphatase 21
Bra039006	2.246	0.052	−5.419	7.14E−23	Retroelement pol polyprotein
Bra022826	12.903	0.408	−4.984	2.66E−56	Triacylglycerol lipase
Bra033779	3.376	0.115	−4.882	6.72E−08	HPP integral membrane domain−containing
Bra029964	55.449	2.248	−4.624	1.35E−191	2−Oxoglutarate-Fe(II)-dependent oxygenase like
Bra005140	118.770	5.278	−4.492	0	Laccase-4
Bra001912	3.939	0.196	−4.331	1.54E−28	Alcohol oxidase-related
Bra026378	3.795	0.222	−4.097	1.68E−23	COBra-like protein 11 precursor
Bra007991	1150.764	85.857	−3.745	0	Sucrose transporter SUC1
Bra031453	1.928	0.219	−3.137	4.76E−07	Serine carboxypeptidase S10 family protein
Bra020052	14.631	2.603	−2.491	2.66E−53	Plasma membrane sulfate transporter
Bra015685	7.629	1.655	−2.205	3.7E−19	Sugar transporter
Bra036819	11.142	3.174	−1.812	2.07E−09	Putative ROP family GTPase
Bra033251	24.180	7.172	−1.753	2.46E−50	CYP86A4
Bra032722	13.487	4.457	−1.598	2.09E−25	Phosphoinositide phospholipase
Bra039028	75.548	28.242	−1.420	8.75E−108	Nucleotide-diphospho-sugar transferase like
Bra013701	3.431	1.362	−1.333	5.71E−04	F−box family protein
Bra000531	16.656	7.927	−1.071	1.35E−13	MYB81
Bra012414	24.322	11.883	−1.033	8.74E−06	Bet v I allergen family protein

### miRNA-target network underlying Ogura-CMS

**Figure 5:**
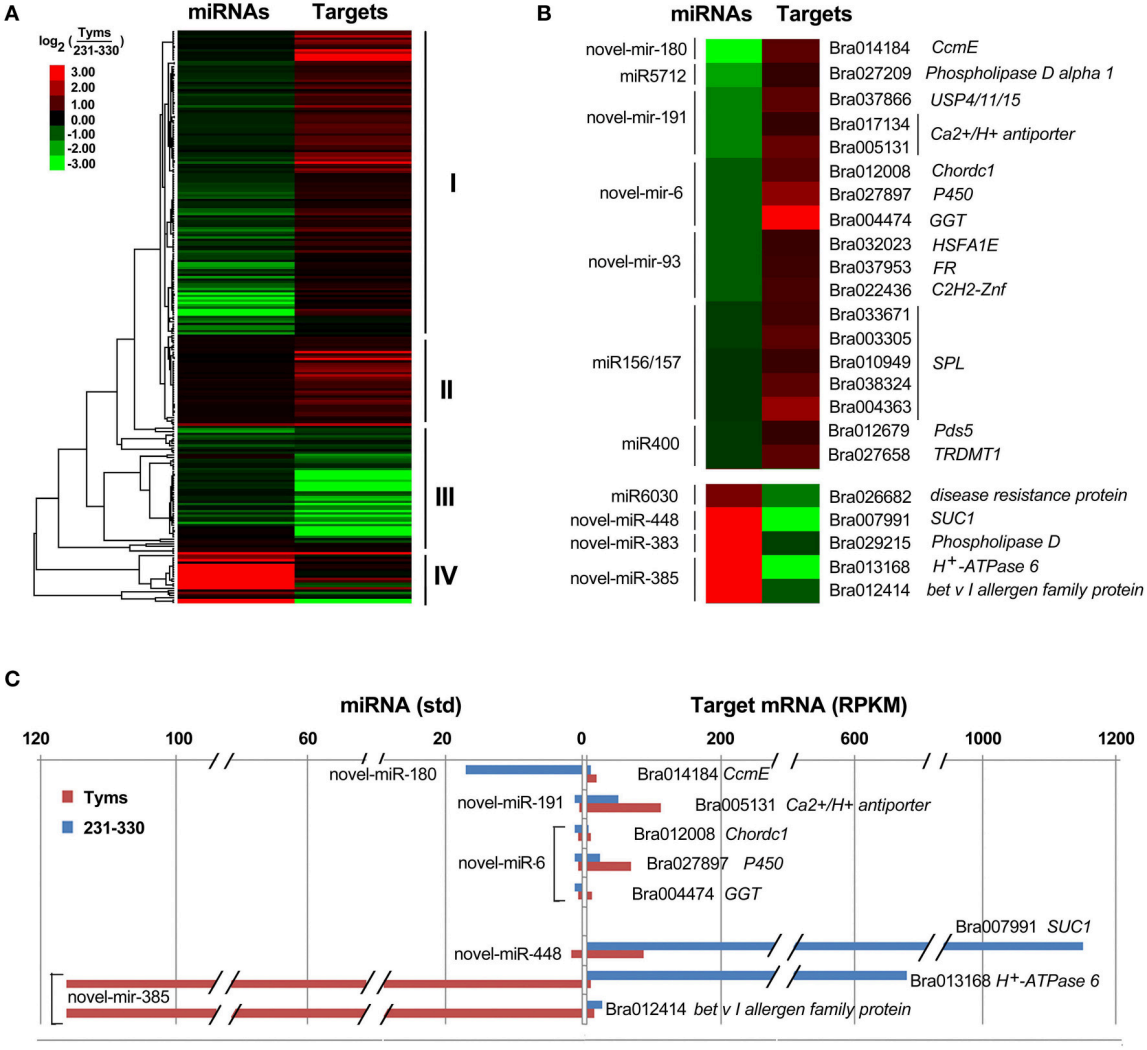
**Expression profiles of miRNA-targets. (A)** Heatmap of overall expression patterns of miRNA-targets. **(B)** Heatmap of expression levels of miRNAs and their targets with more than 1.5-fold changes between Tyms and 231–330. **(C)** Bar chart of expression levels of miRNAs and their targets with more than two-fold changes between Tyms and 231–330. CcmE, cytochrome c biogenesis protein; USP4/11/15, ubiquitin carboxyl-terminal hydrolase 4/11/15; Chordc 1, cysteine/histidine-rich C1 domain-containing protein; P450, cytochrome P450; GGT, glycogenin glucosyltransferase; FR, ferric reduction oxidase 7; C2H2-Znf, C2H2 zinc finger protein 1; SPL, SQUAMOSA promoter binding protein-like; Pds5, sister chromatid cohesion protein PDS5; TRDMT1, tRNA (cytosine38-C5)-methyltransferase; SUC1, sucrose transporter.

**Figure 6:**
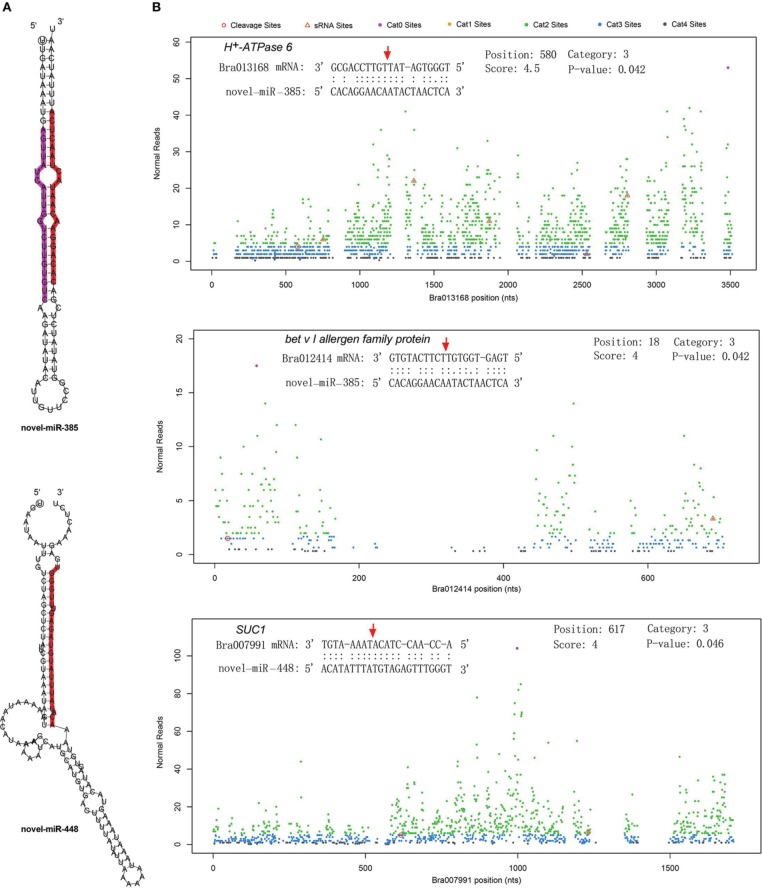
**Secondary structure of two novel miRNAs and the cleavage plots of their targets. (A)** Secondary structure of pre-miRNA hairpins. Red shaded areas indicate the dominant mature miRNAs, violet shaded areas indicates the reverse complementary mature miRNAs. **(B)** Cutting plots of miRNA targets confirmed using degradome sequencing. The corresponding miRNA:mRNA alignments are shown on the top. The red arrows indicate the miRNA-directed cleavage positions. The y-axis shows the nucleotide position in the target gene. The x-axis indicates the number of cleaved ends detected in the degradome analysis.

**Figure 7:**
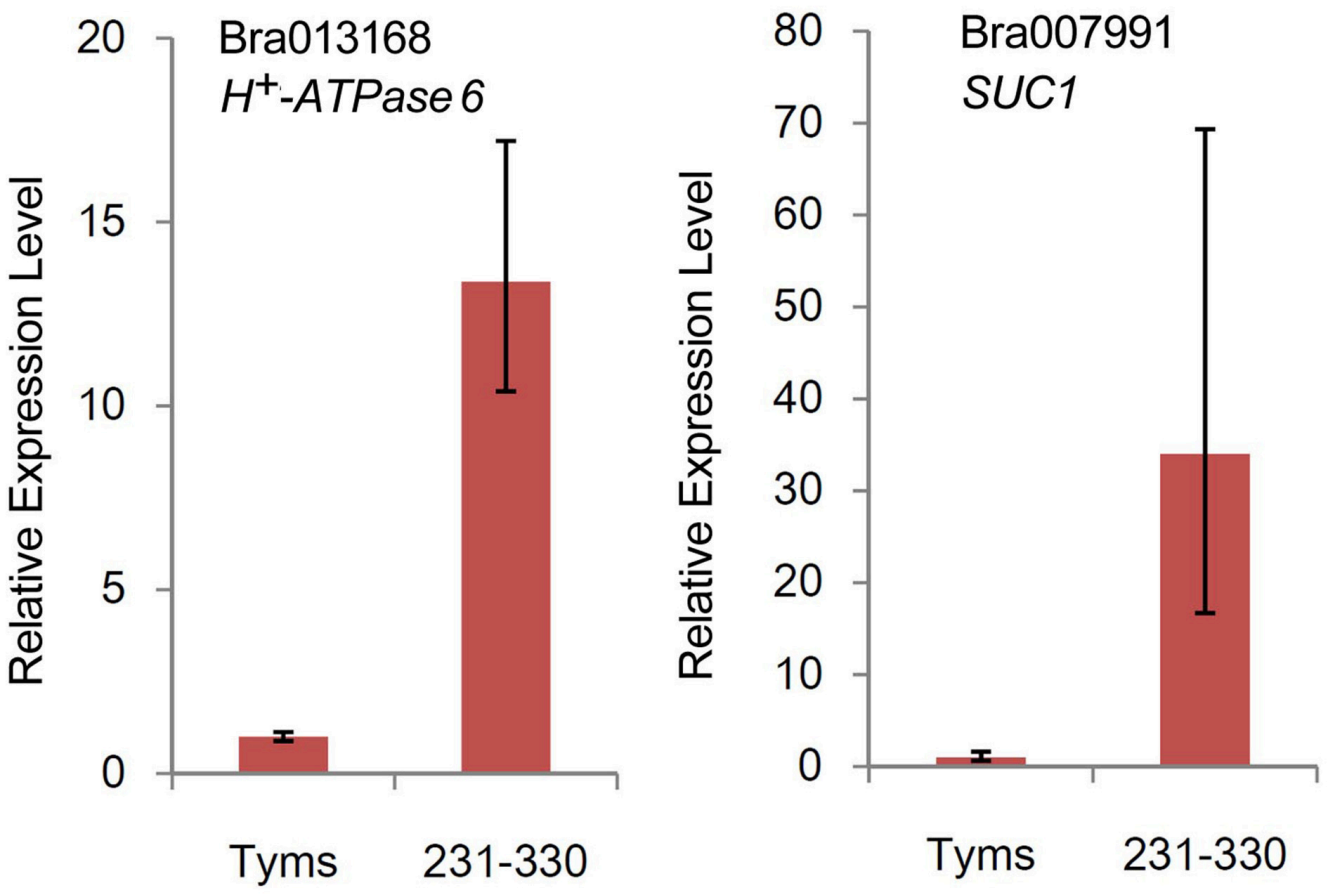
**Relative expression levels of two target genes revealed by Q-PCR**.

The differentially expressed target genes were targeted by 10 known miRNAs (miR159, miR164, miR171, miR172, miR393, miR396, miR397, miR1885, miR5654, and miR6034) and 33 novel miRNAs. However, none of these known miRNAs were significantly differentially expressed according to the two-fold criteria. Based on the combined analysis of the expression profiles of miRNAs and their targets without the significant test filters, the expression patterns of the miRNA-targets could be classified into four clusters. As shown in Figure 5A, cluster I contained more than half of the miRNA-target pairs, in which miRNAs were down-regulated and the targets were up-regulated in Tyms. Approximately 16 and 24% of the miRNA-target pairs were synergistically down-regulated (cluster II) or up-regulated (cluster III), respectively. Less than 8% of the miRNAs were up-regulated while their targets were down-regulated. These results indicate the miRNAs provide an efficient buffer system for fine-tuning the expression of genes involved in bud and pollen development. When a cut-off of >1.5-fold change was used, seven miRNAs were down-regulated and released the expression of 18 targets in Tyms. In contrast, four miRNAs were up-regulated and suppressed the expression of five targets (Figure 5B). miR156 and *SPLs* represent a cascade of controls for many important developmental processes, including the proper development of sporogenic tissues of the anther (Xing et al., 2010). miR156 and *SPLs* are expressed in high abundance in many tissues, including buds (Zhang et al., 2011b). Our present study found that miR156 and *SPLs* were down- or up-regulated in Tyms buds by more than 1.5-fold but less than two-fold. It is possible that the expression of these genes was changed more significantly in sporogenic tissues but was submerged by the expression in other tissues of the buds. Using a two-fold threshold, three and two novel miRNAs were up-regulated in 231–330 and Tyms, resulting in the down-regulation of five and three of their target genes in the corresponding tissues, respectively (Figure 5C). Among them, a cytochrome c biogenesis protein was targeted by novel-miR-180, a Ca^2+^/H^+^ antiporter was targeted by novel-miR-191, a P450 and a glycogenin glucosyltransferase (GGT) were targeted by novel-miR-6; all of these genes have been implicated in pollen development by previous studies (Balk and Leaver, 2001; Welchen and Gonzalez, 2005; Morant et al., 2007; Song et al., 2009; Li et al., 2010, 2012; Rennie et al., 2014) and were up-regulated in Tyms in our analysis. Interestingly, novel-miR-448 and novel-miR-335 were specifically expressed in Tyms; this is especially worth noticing because the novel-miR-335 was expressed at a relatively high level. The expression of its target genes (*sucrose transporter SUC1* and *H*^+^*-ATPase 6*) was high in 231–330 but suppressed by approximately 100-fold in Tyms. SUC1 is an enzyme that catalyzes the degradation of sucrose and performs important roles for pollen germination in *Arabidopsis* (Sivitz et al., 2008). Thus, the down-regulation of *SUC1* may result in energy deficiency in Tyms buds and thus abort pollen development. A study in *Nicotiana plumbaginifolia* reported that co-suppression of an H^+^-ATPase impaired sucrose translocation and male fertility (Zhao et al., 2000). In our present study, both a *SUC1* and an *H*^+^*-ATPase* were indicated as targets of two novel miRNAs and were sufficiently suppressed by these two miRNAs in the Ogura-CMS buds (Figure 5C). This finding indicated that the novel-miR-335/*H*^+^*-ATPase* and novel-miR-448/*SUC1* cascade could play important roles in male sterility in Ogura-CMS. The secondary structure of the novel-miR-335 and novel-miR-448 and the degradation map of *H*^+^*-ATPase* and *SUC1* were shown in Figure 6. The suppression of *H*^+^*-ATPase* and *SUC1* expression were validated by Q-PCR analysis (Figure 7). This finding filled a gap within the cross-talk network between the orf138 locus in mitochondria and the effecter genes in the chromosome. However, more studies are still needed to linkup the orf138 locus and miRNA regulatory networks.

## Conclusion

The present study used deep sequencing technology and performed a series of whole genome small RNA, degradome, and transcriptome analyses on Chinese cabbage buds from both Ogura-CMS and its maintainer. A total of 289 (69 families) known and 426 novel miRNAs were identified, which was much higher than the number of miRNAs found in the buds of the closely relative subspecies *B. campestris* ssp. *chinensis* (Jiang et al., 2014). These miRNAs not only validated the finding in the previous study on *B. campestris* ssp. *chinensis* but also contained many novel miRNAs that were not previously reported. A large number of targets were firstly validated in this study. The combinational profiling of miRNAs and targets revealed a regulatory network contributing to bud development, especially for pollen engenderation. The finding of two novel miRNA/target cascades (novel-miR-335/*H*^+^*-ATPase* and novel-miR-448/*SUC1*) will provide new insights into the communication between the mitochondria and chromosome and take one step toward filling in the gap in the regulatory network mechanism from the orf138 locus to the pollen abortion in the Ogura-CMS plants. The true functions of these two miRNAs warrant more solid experiments, such as the transgenic complementary test.

## Author contributions

XW and XWZ designed the study. XW performed the experiments. XW and XHZ analyzed the data and drafted the manuscript. YY and QZ assisted with the bioinformatics analysis and aided in writing the manuscript. QY, ZW, YZ, and WJ aided in performing the experiments. XL and FW modified the manuscript. All of the authors carefully checked and approved this version of the manuscript.

## Data access

RNAseq are submitted to EMBL/NCBI/SRA with the accession numbers SRR2132359, SRR2136647, SRR2149955, SRR2132463, SRR2136646, SRR2149956.

### Conflict of interest statement

The authors declare that the research was conducted in the absence of any commercial or financial relationships that could be construed as a potential conflict of interest.
